# Production of Putrescine and Cadaverine by *Paucilactobacillus wasatchensis*

**DOI:** 10.3389/fmicb.2022.842403

**Published:** 2022-03-03

**Authors:** Hélène Berthoud, Daniel Wechsler, Stefan Irmler

**Affiliations:** Agroscope, Bern, Switzerland

**Keywords:** *Paucilactobacillus wasatchensis*, *Lactobacillus*, putrescine, cadaverine, ornithine decarboxylase, lysine decarboxylase, cheese, biogenic amines

## Abstract

Lactic acid bacteria (LAB) play a key role in many food fermentations. However, some LAB species can also cause food spoilage, e.g., through the formation of biogenic amines. *Paucilactobacillus wasatchensis* is a LAB that causes late gas production in Cheddar cheese, the molecular causes of which are not fully understood. This study reports on the ability of *P. wasatchensis* WDC04 to produce cadaverine and putrescine in broth supplemented with lysine and ornithine, as well as in a model cheese. The raclette-type semi-hard cheese produced with *P. wasatchensis* as an adjunct culture contained 1,085 mg kg^−1^ of cadaverine and 304 mg kg^−1^ of putrescine after 120 days of ripening. We identified two ornithine decarboxylase genes (*odc*) and a putrescine-ornithine antiporter gene *(potE)* in the genome sequence of *P. wasatchensis*. We could show that the two *odc* genes, which are located on two contigs, are contiguous and form the genetic cluster *odc2-odc1-potE*. Alignment searches showed that similar gene clusters exist in the genomes of *Levilactobacillus paucivorans* DSMZ22467, *Lentilactobacillus kribbianus* YH-lac9, *Levilactobacillus hunanensis* 151-2B, and *Levilactobacillus lindianensis* 220-4. More amino acid sequence comparisons showed that Odc1 and Odc2 shared 72 and 69% identity with a lysine and ornithine decarboxylase from *Ligilactobacillus saerimneri* 30a, respectively. To clarify the catalytic activities of both enzymes, the *odc*-coding genes were cloned and heterologously expressed as His-tagged fusion protein. The purified Odc1 protein decarboxylated lysine into cadaverine, while the recombinant Odc2 protein preferentially produced putrescine from ornithine but also exhibited low lysine decarboxylating activity. Both enzymes were active at pH of 5.5, a value often found in cheese. To our knowledge, this is only the second lysine decarboxylase in LAB whose function has been verified. The tandem arrangement of the genes in a single cluster suggests a gene duplication, evolving the ability to metabolize more amino. Divergent substrate preferences highlight the necessity of verifying the functions of genes, in addition to automatic annotation based on sequence similarity. Acquiring new biochemical data allows better predictive models and, in this case, more accurate biogenic amine production potential for LAB strains and microbiomes.

## Introduction

Fermented foods are defined as foods or beverages produced through controlled microbial growth and the conversion of food components through enzymatic action (Dimidi et al., 2019). Their effect on health is considered positive, despite very limited clinical evidence for such a claim (Gille et al., 2018; Dimidi et al., 2019). Fermentation is also used to enhance organoleptic properties. Cheeses made with raw milk have been shown to contain greater microbial diversity, ripen faster, and develop a richer and more intense flavor than pasteurized milk cheeses (Montel et al., 2014; Egger et al., 2021). However, fermentation may also produce undesired metabolites, such as biogenic amines. The most important BAs in cheese are histamine and tyramine (produced *via* the enzymatic decarboxylation of histidine and tyrosine, respectively), putrescine (synthesized *via* ornithine decarboxylation or agmatine deamination), and, more rarely, cadaverine (originated by lysine decarboxylation; Linares et al., 2011). In hard and semi-hard cheeses, the accumulation of histamine is generally associated with *Lentilactobacillus parabuchneri* (O’Sullivan et al., 2015; Berthoud et al., 2017; Tofalo et al., 2019). Enterococci are considered tyramine formers, but other lactobacilli, such as *Latilactobacillus curvatus* and *Levilactobacillus brevis*, have also been shown to produce high levels of tyramine (Cid et al., 2008; Coton and Coton, 2009; Schirone et al., 2013). Enterobacteria are known to produce cadaverine and putrescine (Coton et al., 2012; Schirone et al., 2012); however, their population density typically decreases considerably in hard and semi-hard cheeses within the first 90 days of ripening (Bachmann and Spahr, 1995; Maher et al., 2001; Rios et al., 2020). In the core of an uncooked pressed type model cheese inoculated with Gram-negative cadaverine producers, only low amounts of cadaverine were measured (<100 mg kg^−1^ cheese dry matter; Delbès-Paus et al., 2012). Regarding the extreme levels of cadaverine (>1,000 mg kg^−1^) reported by Schirone et al. (2013) and Benkerroum (2016) in Pecorino di Fossa, a semi-hard cheese, other microorganisms may be responsible (Linares et al., 2011).

Lactic acid bacteria (LAB) include many species, and some of them are able to grow in cheese during ripening. Moreover, some strains have been shown to produce biogenic amines and contain amino acid-decarboxylating genes (Cid et al., 2008; Romano et al., 2014; Zhang and Ni, 2014; Del Rio et al., 2018). In particular, *Ligilactobacillus saerimneri* 30a was shown to be capable of decarboxylating histidine, ornithine, and lysine into histamine, putrescine, and cadaverine, respectively (Pessione et al., 2005). Romano et al. sequenced the genome and identified a three-component lysine/ornithine decarboxylation system (Romano et al., 2013a,b) composed of an amino acid transporter (PotE; RefSeq WP_009553966) that had affinity for both the lysine/cadaverine and ornithine/putrescine pairs, an ornithine decarboxylase (Odc; RefSeq WP_009553942) with an additional lower affinity for lysine, and a new specific lysine decarboxylase (Ldc; RefSeq WP_009553967). In fact, Odc and Ldc shared 59.4% amino acid identity and short conserved sequences. Previously, the authors also described putative arginine/ornithine/lysine decarboxylases (Aoldc) that were widespread in LAB and were not associated with an ornithine/putrescine exchanger. The Aoldc proteins from *Lacticaseibacillus paracasei* (RefSeq WP_011674560) and *Lactobacillus gasseri* (RefSeq WP_011679015) had a neutral pH optimum but very low activities with ornithine and lysine as substrates. In *L. saerimneri*, Aoldc (RefSeq WP_040533848) shared 49.4 and 47.4% amino acid identity with Ldc and Odc, respectively, as well as having short conserved domains. The similarities between the different decarboxylase proteins make automatic annotations and function predictions tricky. However, in performing a BLAST search using the amino sequence of this lysine decarboxylase as query, one interesting hit was the sequence RefSeq WP_044011228 from *Paucilactobacillus wasatchensis*, which was annotated as ornithine decarboxylase and shared 72% identity with the Ldc of *L. saerimneri*.

Additionally, *P. wasatchensis* sp. nov. was isolated from aged Cheddar (Oberg et al., 2016). This bacterium is a slow-growing obligatory heterofermentative lactobacillus that grows at storage temperatures between 6 and 12°C and up to 30°C. Its growth and survival characteristics allow it to grow in cheese (McMahon et al., 2020), and it was shown to be associated with late gas production in Cheddar cheese during storage (Ortakci et al., 2015b).

The aims of this study were to (i) investigate the capacity of *P. wasatchensis* WDC04 (DSM 29958) to produce cadaverine and putrescine by decarboxylating lysine and ornithine in culture medium, as well as in a trial cheese; (ii) identify the putative amino acid decarboxylase genes; and (iii) evaluate the decarboxylation activity of recombinant proteins, as well as their substrate preference. A raclette-type semi-hard model cheese with adjunct culture of *P. wasatchensis* was produced. After 120 ripening days, the composition of the free amino acids and the biogenic amine content were determined. The quantification of *P. wasatchensis* in cheese was performed by a TaqMan real-time PCR assay designed based on the DNA recombinase A *recA* gene.

## Materials and Methods

### Growth Conditions and Formation of Cadaverine and Putrescine

*P. wasatchensis*, formerly *Lactobacillus wasatchensis*, DSM 29958 obtained from the German Collection of Microorganisms and Cell Cultures GmbH (DSMZ, Braunschweig, Germany) was cultivated in modified MRS broth (mMRS) at 25°C. This medium was prepared according to the cultivation conditions recommended by the DSMZ. This means that MRS (Man et al., 1960) was supplemented with 0.05% L-cysteine and 1.5% D-ribose, and the pH was adjusted to 5.2. To evaluate the capability to produce cadaverine and putrescine, the DSM 29958 strain was incubated in mMRS supplemented with 1% L-lysine, 1% L-ornithine, or 1% L-arginine at 25°C for 4 days. Biogenic amines were derivatized with dansyl chloride, extracted, and separated using high-performance thin-layer chromatography (HPTLC) as described by Li et al. (2016). In contrast to Li et al., the dansylated amines were extracted with ethyl acetate rather than diethyl ether. The solvent system consisted of chloroform-triethylamine (4:1, v:v).

### Protein Sequence Analysis

Database searches were performed using the BLAST programs developed by Altschul et al. (1997). Sequences were aligned with CLUSTAL Omega (1.2.4; Sievers et al., 2011). The alignment was employed to generate and visualize an unrooted tree using SEAVIEW 5.0.4 (Gouy et al., 2010), applying the neighbor-joining (NJ) method, and the Kimura two-parameter model (K2P).

### Production of the Model Cheese With an Adjunct Culture of *P. wasatchensis*

A raclette-type semi-hard model cheeses to which *P. wasatchensis* DSM 29958 was added as an adjunct and a control cheese without the strain were manufactured in the experimental cheese dairy at Agroscope (Bern, Switzerland) as described by Dreier et al. (2020). Briefly, the cheeses were produced from 50 L of pasteurized milk using a combination of the mesophilic starter RSW 901 (*Lactococcus lactis* subsp. *lactis*, *L. lactis* subsp. *cremoris*, and *L. lactis* subsp. *diacetylactis*) and the mixed mesophilic/thermophilic starter MK 401 (*L. lactis* subsp. *lactis*, *Lactobacillus delbrueckii* subsp. *lactis*, and *Streptococcus thermophilus*; Liebefeld Kulturen AG, Switzerland). The estimated concentration of the adjunct culture in the milk vat was 10^4^–10^5^ CFU ml^−1^. The cheeses (30 cm in diameter, about 6 kg) were immersed in a 20% (w/w) saline solution (11–13°C, 14 h) and smear-ripened in a maturing cellar (10–11°C, 90–96% relative humidity) for 120 days.

### Chemical Analysis of Cheeses

Free amino acid concentrations were determined using high-performance liquid chromatography (HPLC), as previously described (Wenzel et al., 2018). Biogenic amines were derivatized with dansyl chloride prior to ultra-performance liquid chromatography (UPLC) separation, as previously described (Ascone et al., 2017).

### DNA Extraction and Species-Specific qPCR

The DNA was extracted from a pure culture of *P. wasatchensis*, as well as from the cheeses, as described by Berthoud et al. (2017). For the species-specific quantitative real-time PCR (qPCR), primers and probe were designed based on the gene *rec*A, which codes for the DNA recombinase A protein (RefSeq NZ_AWTT01000028 REGION: 23402.24457). The assays were performed in a reaction volume of 12 μl containing 6 μl of Takyon™ No ROX Probe 2X MasterMix UNG (Eurogentec, Seraing, Belgium), 300 nM of forward (5′-CGAATTGGTCAAGGTCGAGAA-3′) and reverse primer (5′-GACCTTTTGCATTAACTCATCCATT-3′), 100 nM of hydrolysis probe (5′-FAM-TGCCAAACAATATTTCGCTGATCATCCAG-BHQ1-3′), and 2 μl of DNA. The qPCR conditions were 50°C for 2 min and 95°C for 3 min, followed by 40 cycles of 95°C for 3 s and 60°C for 20 s. All qPCR assays were run on a Corbett Rotor-Gene 3000 (Qiagen, Hilden, Germany), and a serial dilution of the genomic DNA of strain DSM 29958 was included in each run. The DNA concentration was determined using a NanoDrop® ND-1000 Spectrophotometer (NanoDrop Technologies, Thermo Fisher Scientific, Waltham, MA, United States). The number of copies, corresponding to DNA concentration, was estimated using 660 pmol pg^−1^ as the average molecular weight for a nucleotide pair and 1.90425 Mb as the genome size of *P. wasatchensis*. The analysis was performed using Rotor-Gene Q Series Software v2.3.1 (Qiagen), using a threshold of 0.05 for the quantification cycle (Cq) value determination.

### Cloning of Putative Decarboxylases Genes *odc*1 and *odc*2

The genes coding for the putative ornithine decarboxylases *odc*1 and *odc*2 from *P. wasatchensis* were amplified with primers containing a restriction site for *Bam*HI and *Sal*I (restriction sites are underlined). The primers were designed using the sequences of the *odc*1 gene (odc1_F 5′-ATATGGATCCAATGAAATTATTAGGTATCGCGT-3′; odc1_R 5′-TCGAGTCGACCGTTTTTCTTTATTACAGATTCATCA-3′) and odc2 gene (odc2_F 5′-ATATGGATCCAATGAATAGTATGAAAATTGCTACAA-3′; odc2_R 5′-TCGAGTCGACCTTTTTGCTCTTCTAGCACGTA-3′). Amplification reactions were performed in a final volume of 50 μl, containing 25 μl of 2× Platinum™ SuperFi™ PCR Master Mix (Invitrogen, Thermo Fisher Scientific), 2.5 μl of each primer at 0.5 mM, and 25 ng of genomic DNA. Amplifications were carried out in a thermocycler (Applied Biosystems, Thermo Fisher Scientific) with the following program: initial denaturation at 98°C for 30 s, followed by 5 cycles at 98°C for 10 s, 52°C for 10 s, and 72°C for 2 min; then, there were 30 cycles under the same conditions but with an annealing temperature of 65°C, as well as a final prolongation of 5 min at 72°C. The DNA fragments obtained were digested with enzymes and then ligated with T4 DNA ligase (Invitrogen) into the pEG-His1 (MoBiTec Molecular Biotechnology, Goettingen, Germany) vector previously digested with the same enzymes. One-Shot™ Mach1 chemically competent *E. coli* (Invitrogen) was transformed with the ligation product. The positive clones obtained were named Odc1_Lw35 and Odc2_C19, respectively.

### Production and Purification of Recombinant Proteins

The expression plasmids were transformed into One-Shot™ BL21(DE3) chemically competent *E. coli* (Invitrogen) according to the manufacturer’s instructions. The transformation reaction was plated into LB agar plates containing 0.1 mg/ml ampicillin. After incubation overnight at 37°C, a colony was transferred into 100 ml of MagicMedia™ *E. coli* expression medium (Thermo Fisher Scientific). The medium was then incubated at 30°C for 20 h on a shaker (220 rpm).

The bacterial cells were harvested *via* centrifugation (3,000 *g*, RT, 10 min), washed once with 20 mM of sodium phosphate (pH 7.4), and frozen at −20°C. The recombinant, his-tagged protein was purified using a Protino Ni-TED 1000 Kit (Machery-Nagel, Oensingen, Switzerland) according to the manufacturer’s protocol. The buffer of the eluate containing the recombinant protein was exchanged with 20 mM of sodium phosphate (pH 7.4) using illustra™ NAP™ columns (VWR International GmbH, Dietikon, Switzerland) according to the manufacturer’s protocol. The protein concentration was determined using the Qubit Protein Assay Kit (Thermo Fisher Scientific). The purity of the purified proteins was evaluated using denaturing polyacrylamide gel electrophoresis, followed by Coomassie Blue staining.

### Decarboxylase Activity Assay

The above mentioned methods to determine biogenic amines qualitatively by HPTLC or quantitatively by HPLC cannot be used to detect amino acids. In order to observe both the amino acid substrates (lysine and ornithine) and amine (cadaverine and putrescine), a third method was used here. O-phthalaldehyde was used to derivatize amino acid and amine which were then separated and quantified by HPLC as previously described (Wenzel et al., 2018). This allowed to control that the substrate was not completely used up during the enzymatic reaction. The decarboxylation assay was performed in a volume of 200 μl. The assay consisted of 50 mM phosphate buffer (pH 5.5), 50 μM of pyridoxal 5′-phosphate, and 10 mM of amino acid. L-Lysine, L-ornithine, and L-arginine were used as amino acids. The reaction was performed with 2 μg of purified protein at 37°C for 30 min. The exception was the decarboxylation of ornithine by Odc2_C19, for which only 0.2 μg of enzyme was used and the reaction time was 10 min.

For the determination of the kinetic parameters, the substrate concentration varied between 1 and 50 mM. Phosphate buffers with different pHs were used to determine the dependence on pH.

The enzymatic reaction was stopped by adding 200 μl of methanol. The sample was then filtered through a 0.45 μm filter (nylon). The amines present in the filtrate were derivatized with O-phthalaldehyde and separated *via* HPLC, as described previously (Wenzel et al., 2018). Calibration standards were prepared with putrescine and cadaverine in 50% methanol. The peak areas of putrescine and cadaverine were determined with Chromeleon software (v7.2.10, Thermo Fisher Scientific). The experimental data were fitted to the Michaelis–Menten equation using SciPy (Virtanen et al., 2020) to determine the Km and kcat.

## Results

### Formation of Biogenic Amines by Strain DSM 29958

**Figure 1:**
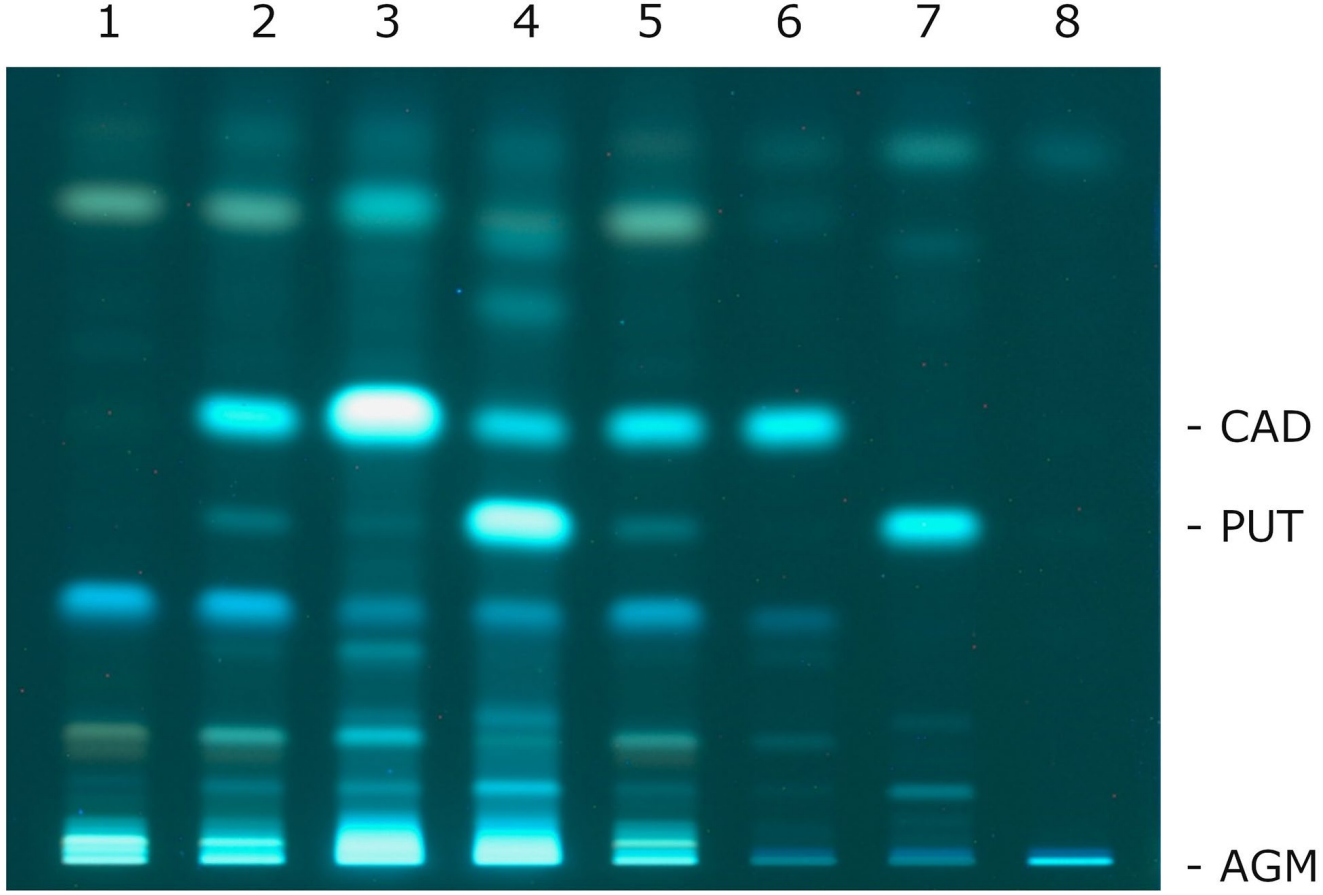
Thin-layer chromatography of the biogenic amines presents in the culture supernatants of *P. wasatchensis*. 1: uninoculated medium; 2: medium; 3: medium + lysine; 4: medium + ornithine; 5: medium + arginine; 6: cadaverine; 7: putrescine; and 8: agmatine.

Cadaverine and putrescine were detected in all culture supernatants (Figure 1). Neither compound was present in the uninoculated medium. Upon the addition of lysine and ornithine, increased formations of cadaverine and putrescine, respectively, were observed. Medium without the addition of lysine allowed the production of cadaverine, probably due to low levels of lysine in the MRS broth. When the medium was supplemented with arginine, neither agmatine nor putrescine formation was observed.

### Determination of Biogenic Amines, Free Amino Acids, and *P. wasatchensis* in Model Cheeses

The concentrations of biogenic amines (cadaverine, putrescine, histamine, and tyramine) and their amino acid precursors (lysine, ornithine, histidine, and tyrosine) were measured in a model cheese inoculated with *P. wasatchensis*, as well as in a control cheese, after 120 ripening days. Cadaverine and putrescine were detected at 1,085 and 304 mg kg^−1^ in cheese with *P. wasatchensis*, while the amounts of the respective amino acid precursors were reduced in comparison with the control cheese. No histamine and negligible concentrations of tyramine were detected. Amounts of histidine and tyrosine, as well as total free amino acids (FAAs), were similar in both cheeses (Table 1). *P. wasatchensis* was measured *via* qPCR in the inoculated cheese at an estimated concentration of 1.16 × 10^7^ genome equivalents (GE) per gram, while no amplification signal was detected in the control cheese (Table 1). These results showed the concomitant growth of *P. wasatchensis*, the production of cadaverine and putrescine, as well as a reduction of the levels of lysine and ornithine as compared to the control cheese.

**Table 1:** Concentrations of biogenic amines (mg kg^−1^), free amino acids (FAAs, mmol kg^−1^), and *P. wasatchensis* (genome equivalent per g; GE g^−1^) in model cheeses ripened for 120 days.

	Biogenic amines (mg kg^−1^)				Free amino acid (mmol kg^−1^)					*P. wasatchensis* (GE g^−1^)
	CAD	PUT	HIST	TYRA	Lys	Orn	Hist	Tyr	Total FAAs	
Cheese with *P. wasatchensis*	1,085	304	ND	<LOQ	1.05	0.26	2.82	3.16	193	7 log
Control cheese	<LOQ	ND	ND	<LOQ	12.74	3.87	3.8	3.62	208	ND

ND, not detected; LOQ, limit of quantitation; CAD, cadaverine; PUT, putrescine; HIST, histamine; TYRA, tyramine; Lys, lysine; Orn, ornithine; Hist, histidine; and Tyr, tyrosine.

### Identifying and Cloning Two Putative Ornithine Decarboxylase Genes

The genome sequence of *P. wasatchensis* strain WDC04 is deposited in the NCBI’s Reference Sequence database (RefSeq NZ_AWTT00000000.1). This data entry contains 1773 proteins. It contains one complete ornithine decarboxylase and two partial ornithine decarboxylases. The complete one (WP_044011228) has a length of 726 amino acids, is located on contig000042, and is referred to as Odc1 in this report. Downstream of Odc1 is a putative putrescine-ornithine antiporter (PotE; WP_044011229). The two partial ornithine decarboxylases are found on contig000080 (WP_044011535) and contig000042 (WP_044011227), respectively. WP_044011227 has a length of 60 amino acids. We suspected that this is the missing part of the protein WP_044011535, which has a length of 660 amino acids, and that an assembly error likely caused the coding sequence to be spread over two contigs. To test this hypothesis, an attempt was made to amplify the junction sequence of both contigs *via* PCR.

**Figure 2:**
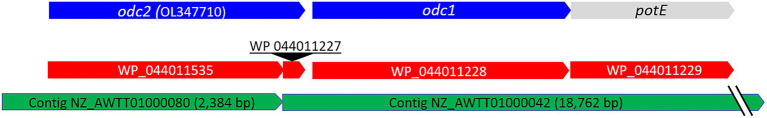
Schematic illustration of the genetic region involved in lysine and ornithine decarboxylation in *P. wasatchensis*. The green and red arrows represent the RefSeq accession numbers of the genomic and protein data, respectively. The blue arrows represent the *odc1* and *odc2* genes that were cloned in this study. The gray arrow represents the ornithine-putrescine antiporter gene *potE*.

Therefore, the primers Lwasa_odc2_F 5′-CTTCCAGAACCAGCAATGACA-3′ and Lwasa_odc1_R 5′-TTAATCAAGTCGAGATCATCTTCAGT-3′ were designed, which have binding sites on contig00080 and contig00042, respectively. The PCR produced an amplicon of about 500 bp as expected, confirming that the contigs are concatenated (data not shown). The complete gene was named *odc*2, and its sequence is deposited in the GenBank database under the accession number OL347710. Figure 2 represents how the two contigs are related, and the genes *odc1*, *odc2*, and *potE*.

### Database Searches and Similarity Trees

**Figure 3:**
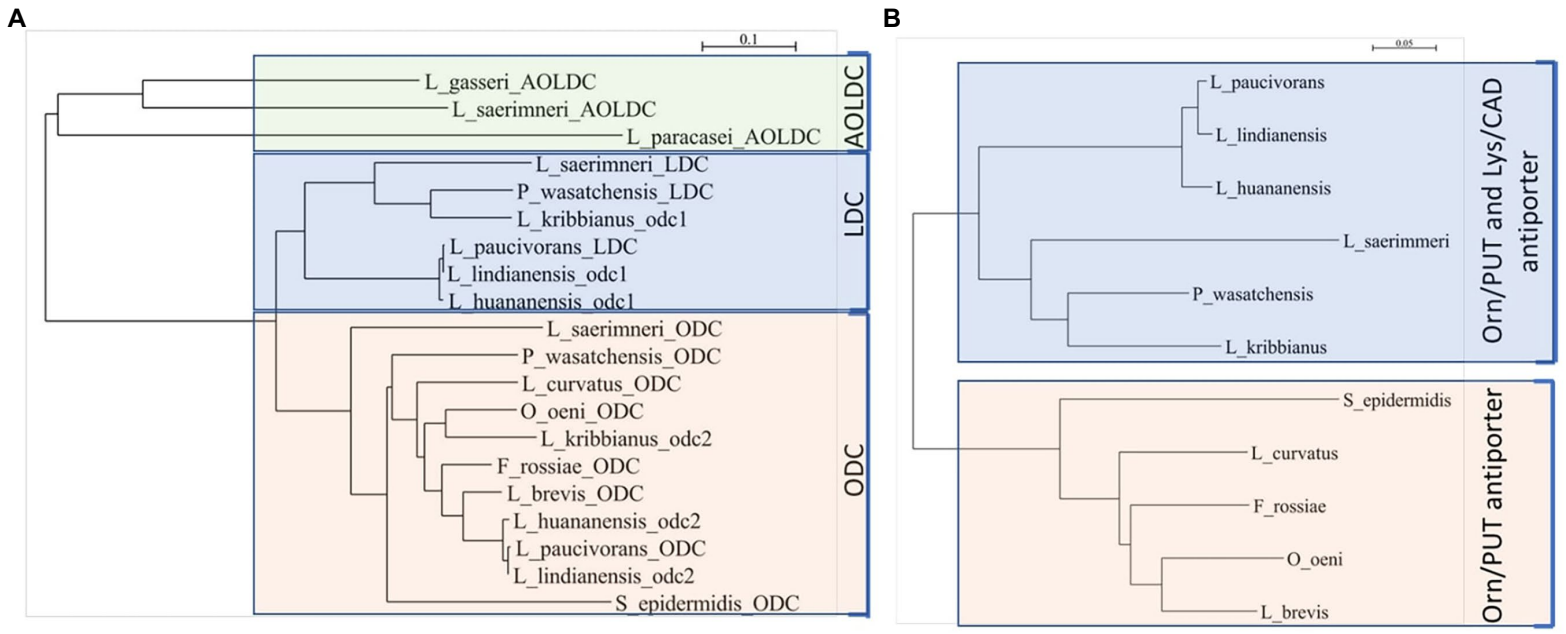
Neighbor-joining trees based upon the alignment of amino acid sequences of representative decarboxylases **(A)** and associated antiporter **(B)** and the predicted function. ODC, ornithine decarboxylase; LDC, lysine decarboxylase; AOLDC, arginine/ornithine/lysine decarboxylase; PUT, putrescine; CAD, cadaverine; Orn, ornithine; and Lys, lysine.

The genetic organization *odc2-odc1-potE* (Figure 2) was found using BLAST searches against the genome sequence of other lactic acid bacteria, such as *Levilactobacillus paucivorans* DSM 22467 (GenBank ASM143712v1), *Levilactobacillus lindianensis* strain 220-4 (GenBank ASM394609v1), *Levilactobacillus hunanensis* strain 151-2B (GenBank ASM394614v1), and *Lentilactobacillus kribbianus* strain YH-lac9 (GenBank ASM1318407v1). To predict the preferred substrates of these putative decarboxylases, protein sequences with biochemically demonstrated functions, that is, ODC for ornithine decarboxylation, LDC for lysine decarboxylation, and AOLDC for the biosynthetic arginine/ornithine/lysine decarboxylation of Gram-positive bacteria, were selected (Table 2) and aligned with the Odc1 and Odc2 mentioned above. Similarities were visualized with a neighbor-joining tree (Figure 3A). The ODC and Odc2 amino acid sequences clustered together and were distinct from LDC and Odc1, which formed another cluster. The AOLDC protein sequences were apart from both the ODC and LDC clusters. Based on these results, we hypothesized that Odc1 from *P. wasatchensis* decarboxylates lysine to cadaverine, while Odc2 produces putrescine from ornithine. PotE is essential for the import of amino acid and the secretion of biogenic amines and is generally located next to the decarboxylase gene. The similarity tree for the reference PotE sequences (Figure 3B) showed two clusters, one with the PotE sequences associated with a unique ODC, as well as another one with PotE sequences associated with both ODC and LDC, such as, for example, *P. wasatchensis*.

**Table 2:** Accession number of reference sequences retrieved from GenBank for phylogenetic analysis.

Organisms	Aoldc	Ldc/odc1	Odc/odc2	PotE	References
*Lacticaseibacillus paracasei*	Q038E5				Romano et al., 2012
*Lactobacillus gasseri*	WP_011679015				Romano et al., 2012
*Ligilactobacillus saerimneri*	WP_040533848	WP_009553967	WP_009553942	WP_009553966	Romano et al., 2013b
*Oenococcus oeni*			CAG34069	CAM07323	Romano et al., 2012
*Furfurilactobacillus rossiae*			ANJ65946	ANJ65947	Del Rio et al., 2018
*Levilactobacillus brevis*			AFC60624	AFC60625	Romano et al., 2012
*Latilactobacillus curvatus*			WP_039099122	WP_039099123	Li et al., 2018, 2019
*Paucilactobacillus wasatchensis*		WP_044011228	OL347710	WP_044011229	this study
*Levilactobacillus paucivorans*		WP_057879136	WP_057879135	WP_057879137	unpublished data
*Staphylococcus epidermidis*			ADJ57328	ADJ57329	Coton et al., 2010
*Levilactobacillus lindianensis*		WP_125546544	WP_125546542	WP_125546546	
*Lentilactobacillus kribbianus*		WP_172188509	WP_172188507	WP_172188511	
*Levilactobacillus hunanensis*		WP_125574408	WP_125574407	WP_125574409	

Only publications describing decarboxylase activity are mentioned under “References.”

### Decarboxylase Activity of Recombinant Proteins

The two genes *odc*1 and *odc*2 were cloned into the expression vector pEG-His1 to study their function in more detail. The Sanger sequences of positive clones Odc1_Lw35 and Odc2_C19 confirmed the correct insertion of both the *odc*1 and *odc*2 genes, respectively. Cloning fused a His-tag to the C-terminus of the putative ornithine decarboxylases, which allowed the recombinant proteins to be purified by Ni-metal affinity chromatography. The *E. coli* expression strain BL21(DE3) also possesses ornithine and lysine decarboxylases. Therefore, the protein extract of *E. coli* BL21(DE3), which did not harbor a plasmid, was also separated by Ni-metal affinity chromatography. Neither ornithine decarboxylation nor lysine decarboxylation were detected in the fraction of this *E. coli* control extract that was eluted from the column material (data not shown).

For the enzyme tests used, it was found that Odc1_Lw35 decarboxylated lysine to cadaverine. No activity was detected for this enzyme with ornithine and arginine. Odc2_C19 decarboxylated both lysine and ornithine and the two products cadaverine and putrescine, respectively, were detected. Odc2_C19 showed no decarboxylating activity toward arginine.

**Figure 4:**
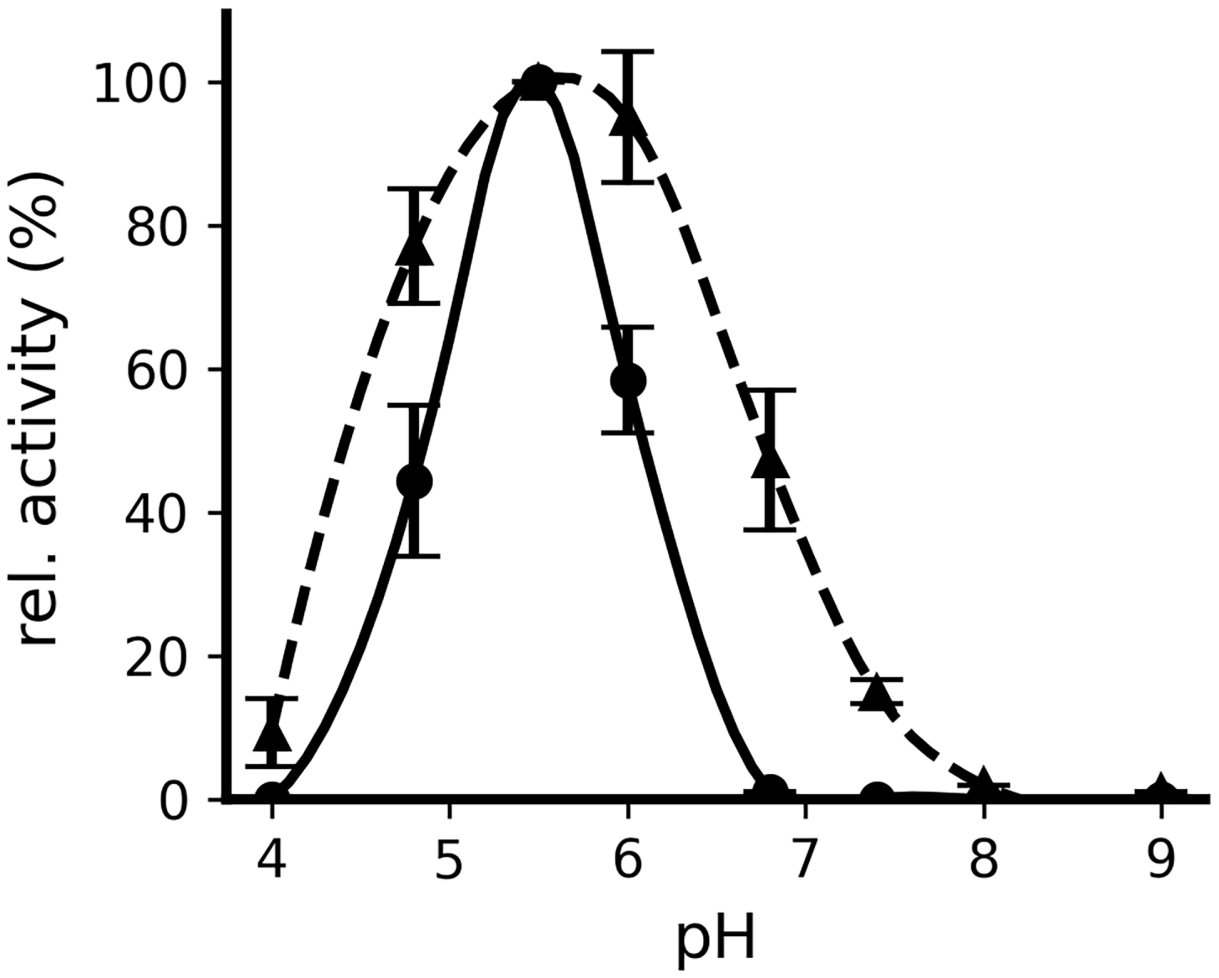
Influence of pH on the decarboxylating activity of Odc1_Lw35 (●) and Odc2_C19 (▲). The relative activity is illustrated with respect to the activity measured at pH 5.5. Odc1_Lw35 was incubated with 10 mM of L-lysine and Odc2_C19 with 10 mM of L-ornithine. The illustration represents the mean (± standard deviation) of triplicates.

When the pH dependence of the decarboxylation reaction was determined for both proteins, Odc1_Lw35 was found to be active in a narrower pH range than Odc2_C19 (Figure 4). Indeed, Odc1_Lw35 showed decarboxylation activity at pH 4.8, 5.5, and 6.0 with an optimum at pH 5.5. Odc2_C19 showed detectable decarboxylation activity from pH 4.0 to pH 7.4 with the highest activity at pH 5.5 and pH 6.0.

The kinetic parameters for both enzymes were determined at pH 5.5, a value that also occurs in cheese. The kinetic parameters for lysine as a substrate show that Odc1_Lw35 has a slightly lower Km value and a slightly higher kcat value than Odc2_C19 (Table 3). When the kinetic parameters of the two substrates ornithine and lysine were compared for Odc2_C19, the enzyme clearly preferred the former.

**Table 3:** Kinetic parameters of Odc1_Lw35 and Odc2_C19.

Enzyme	Substrate	Km (mM)	kcat (s^−1^)
Odc1_Lw35^#^	lysine	16.0 ± 1.8	3.0 ± 0.1
Odc2_C19	lysine	20.2 ± −0.4	1.8 ± 0.1
Odc2_C19	ornithine	4.9 ± 0.9	275 ± 43.37

Values represent the means of triplicates (Km. Michaelis–Menten constant; kcat: turnover number).

# *Odc1: decarboxylating activity toward ornithine was not detected*.

## Discussion

This study shows that the *P. wasatchensis* strain DSM 29958 produces cadaverine and putrescine in media supplemented with lysine and ornithine, respectively. Upon the addition of arginine to the culture medium, neither agmatine nor putrescine were observed, suggesting that there is no arginine decarboxylase activity or arginine deiminase (ADI) pathway to convert arginine into ornithine upstream of putrescine production, as described for the *Weissella halotolerans* strain W22 and *L. rossiae* strain D87 (Pereira et al., 2009; Del Rio et al., 2018). This is in line with the genomic data for DSM 29958, in which no gene is annotated as arginine deiminase.

There are a few points to note regarding agmatine. Figure 1 shows that the dansylated agmatine did not migrate in HPTLC using a solvent system made up with chloroform-triethylamine. This is consistent with the studies of Li et al. (2016) who used different solvent systems consisting of chloroform-triethylamine with and without diethyl ether. For all studied cases, the authors report a retention factor (Rf) value of 0 for agmatine. Romano et al. (2012) describe that they separated agmatine with a solvent system consisting of chloroform-triethylamine-methanol. Unfortunately, this study does not report an Rf value. The solvent system was also used in this study and, indeed, it was observed that the dansylated reference substance agmatine migrated a few millimeters (data not shown). However, it was not possible to clearly separate agmatine from the unknown compounds that also appeared in the culture supernatants (lane 2 in Figure 1). Therefore, the signal intensities of the dansylated compounds that were found in the fermented medium without and with arginine and that had the same Rf values as the dansylated agmatine were compared (for example: lane 2 and lane 5 in Figure 1). Since no clear different signal intensities were found, it was concluded that no agmatine was formed. This conclusion is supported by the absence of a gene encoding an arginine decarboxylase in the genome of *P. wasatchensis*.

Similarly, in trial cheese produced with an adjunct *P. wasatchensis* culture, *P. wasatchensis* could grow to approximately 7 log genome equivalents per gram and produce cadaverine and putrescine, while the amounts of the respective amino acid precursors were reduced in comparison with the control cheese. More cadaverine (1,085 mg kg^−1^) than putrescine (304 mg kg^−1^) was measured, probably because the cheese contained more lysine than ornithine. Because casein does not contain ornithine, this amino acid is supposed to have been produced by *Lactococcus lactis*, which was present in the applied starter culture, *via* the ADI pathway (Fernández and Zúñiga, 2006; Linares et al., 2011). However, the relative amounts of biogenic amines produced could be influenced by other factors, such as pH, redox potential, and the presence of cofactors (De Filippis et al., 2013; Durak-Dados et al., 2020).

*P. wasatchensis* is a spoilage organism associated with late gas production in Cheddar cheese. It produces gas in medium when galactose is present (Ortakci et al., 2015a). From this, it was deduced that the degradation of galactose in cheese can lead to increased gas formation. Indeed, cheese experiments showed that the addition of ribose and galactose in the presence of *P. wasatchensis* caused increased gas formation (Ortakci et al., 2015b). However, in the same study, gas formation was also observed in the cheeses with *P. wasatchensis* without the addition of sugar. Therefore, it can be concluded that other substrates are also responsible for the undesirable gas formation. In the cheese experiment of this study, cadaverine and putrescine were produced. The production of both substances resulted from the decarboxylation of lysine and ornithine, respectively. In can be calculated that the amount of cadaverine (1,085 mg kg^−1^) and putrescine (304 mg kg^−1^) could yield about 14 mmol kg^−1^ of carbon dioxide, which corresponds to approximately 0.31 L kg^−1^ of cheese assuming that the molar volume is 22.4 L at 0°C and 1 atm.

Ortakci et al. (2015b) produced cheese in which galactose was added to the curd before pressing. In this way, the cheeses with the highest galactose content contained 0.238 g kg^−1^ at the beginning of ripening. Using the above mathematical calculation, the metabolization of galactose would produce 0.29 L kg^−1^. In summary, this means that both the degradation of galactose and amino acids can produce gas of the same magnitude. Since the glucose and the galactose resulting from lactose are consumed by the starter culture within the first 24 h in many cheese varieties, it is recommended to also determine the biogenic amines in Cheddar cheese affected by late blowing in future studies. This would give an indication of the extent to which amino acid decarboxylation is involved in the cheese defect.

To better understand the molecular basis of biogenic amine formation, the genomic data of *P. wasatchensis* were analyzed for the presence of genes encoding amino acid decarboxylases. We identified a gene cluster with three contiguous genes, *odc2*-*odc1*-*potE*, which had similarities with the respective genes involved in the three-component lysine/ornithine decarboxylation system in *Ligilactobacillus saerimneri* 30a. The components are an ornithine decarboxylase, a lysine decarboxylase, and a transporter able to exchange lysine for cadaverine as well as ornithine for putrescine (Romano et al., 2012). Concerning *P. wasatchensis*, the heterologously expressed protein Odc1_Lw35 decarboxylated lysine to cadaverine. The recombinant protein Odc2_C19 preferentially produced putrescine from ornithine but could also produce cadaverine from lysine with a lower affinity. This lower affinity for lysine was also observed for the Odc enzyme of *L. saerimneri* 30a by Romano et al. (2013b). The catalytic efficiency of Odc2_C19 was about 600-fold higher with ornithine as compared to lysine, but secondary lysine decarboxylase activity was clearly demonstrated. In the trial cheese, cadaverine was measured after 120 days of ripening, but the contributions of both enzymes cannot be assessed with wild-type bacteria. These contributions could further be investigated if knock-out strains were available.

However, given the contiguous presence of two similar genes with similar functions, one of them conferring an additional side-function identical to the function of the second gene could be a perfect example of the Innovation-Amplification-Divergence (IAD) model (Bergthorsson et al., 2007). This model explains the origin of new genes *via* selection pressure. Concretely, it suggests that, under selection for lysine decarboxylation, gene coding for ornithine decarboxylase with side lysine decarboxylation activity (*odc2*)is duplicated to increase the overall lysine decarboxylation activity. This new copy (*odc1*) evolves to improve LDC activity. This process may be particularly advantageous for adaptation to a new ecological niche, for example, when moving from an ornithine-rich environment to a lysine-rich one. The ability to decarboxylate lysine to cadaverine could allow for better resistance to an acidic pH, as demonstrated for ornithine decarboxylation by *L. rossiae* (Del Rio et al., 2018), or to osmotic stress through tyrosine decarboxylation by *Streptococcus thermophilus* (La Gioia et al., 2011). Finally, the decarboxylation of amino acids as an alternative energy source is often mentioned but has not been universally demonstrated (Cid et al., 2008; Del Rio et al., 2018). Possessing genes coding for amino acid decarboxylation seems to be advantageous, but such genes are generally not universally distributed in a species. Guerrini et al. (2002) reported that 16% of *Oenococcus oeni* had the capability to form ornithine. Evidence of horizontal transfers was reported (Coton and Coton, 2009; La Gioia et al., 2011; Romano et al., 2014; Wüthrich et al., 2017; Del Rio et al., 2018). It would be interesting to evaluate the potential of other *P. wasatchensis* strains to form biogenic amines. In a general sense, screening bacteria for their potential diamines production could be made more successful by searching for the *potE* gene because this avoids the detection of constitutive decarboxylases (Aoldc). The substrate affinity of decarboxylases must be determined biochemically. However, with pheno- and genotypic data from more strains, substrate specificity prediction should become more precise.

In summary, this study showed the ability of *P. wasatchensis* to produce both cadaverine and putrescine in culture medium and in a trial cheese. Lysine decarboxylase genes of lactic acid bacteria could explain very high amounts of cadaverine (>1,000 mg kg^−1^) in cheese or other fermented products and should be further investigated.

## Data Availability Statement

The datasets presented in this study can be found in online repositories. The names of the repository/repositories and accession number(s) can be found at: https://www.ncbi.nlm.nih.gov/genbank/, OL347710.

## Author Contributions

HB, DW, and SI contributed to conception and design of the study. DW designed the cheese trial. SI performed the biochemical analysis and wrote sections of the manuscript. HB performed the molecular biology analysis and wrote the first draft of the manuscript. All authors contributed to the article and approved the submitted version.

## Conflict of Interest

HB, DW, and SI were employed by Agroscope.

## Publisher’s Note

All claims expressed in this article are solely those of the authors and do not necessarily represent those of their affiliated organizations, or those of the publisher, the editors and the reviewers. Any product that may be evaluated in this article, or claim that may be made by its manufacturer, is not guaranteed or endorsed by the publisher.
